# Supplementary material for: The Toxicological Assessment of Anoectochilus burmannicus Ethanolic-Extract-Synthesized Selenium Nanoparticles Using Cell Culture, Bacteria, and Drosophila melanogaster as Suitable Models
Source: Nanomaterials (Basel). 2023 Oct 22;13(20):2804. doi: 10.3390/nano13202804 (PMC10609996; doi:10.3390/nano13202804)
Supplement: Supplementary file 1 [file nanomaterials-13-02804-s001.zip › nanomaterials-2674228-supplementary.pdf]

The Toxicological Assessment of *Anoectochilus burmannicus* Ethanolic-Extract-Synthesized Selenium Nanoparticles Using Cell Culture, Bacteria, and *Drosophila melanogaster* as Suitable Models

Pensiri Buacheen [1,2], Jirarat Karinchai [2], Woorawee Inthachat [3], Chutikarn Butkinaree [4], Chonchawan Jankam [4], Ariyaphong Wongnoppavich [2], Arisa Imsumran [2], Teera Chewonarin [2], Nuttaporn Pimpha [5], Piya Temviriyanukul [3] and Pornsiri Pitchakarn [2,*]

[1] PhD Program in Biochemistry, Faculty of Medicine, Chiang Mai University, Chiang Mai 50200, Thailand; pensiri_bua@cmu.ac.th

[2] Department of Biochemistry, Faculty of Medicine, Chiang Mai University, Chiang Mai 50200, Thailand;
jirarat.ka@cmu.ac.th (J.K.); ariyaphong.w@cmu.ac.th (A.W.); arisa.bonness@cmu.ac.th (A.I.); teera.c@cmu.ac.th (T.C.)

[3] Food and Nutrition Academic and Research Cluster, Institute of Nutrition, Mahidol University, Nakhon Pathom 73170, Thailand; woorawee.int@mahidol.ac.th (W.I.); piya.tem@mahidol.ac.th (P.T.)

[4] National Omics Center, National Science and Technology Development Agency, Pathum Thani 12120, Thailand; chutikarn.but@nstda.or.th (C.B.); chonchawan.jan@ncr.nstda.or.th (C.J.)

[5] National Nanotechnology Center, National Science and Technology Development Agency, Thailand Science Park, Pathum Thani 12120, Thailand; nuttaporn@nanotec.or.th

* Correspondence: pornsiri.p@cmu.ac.th

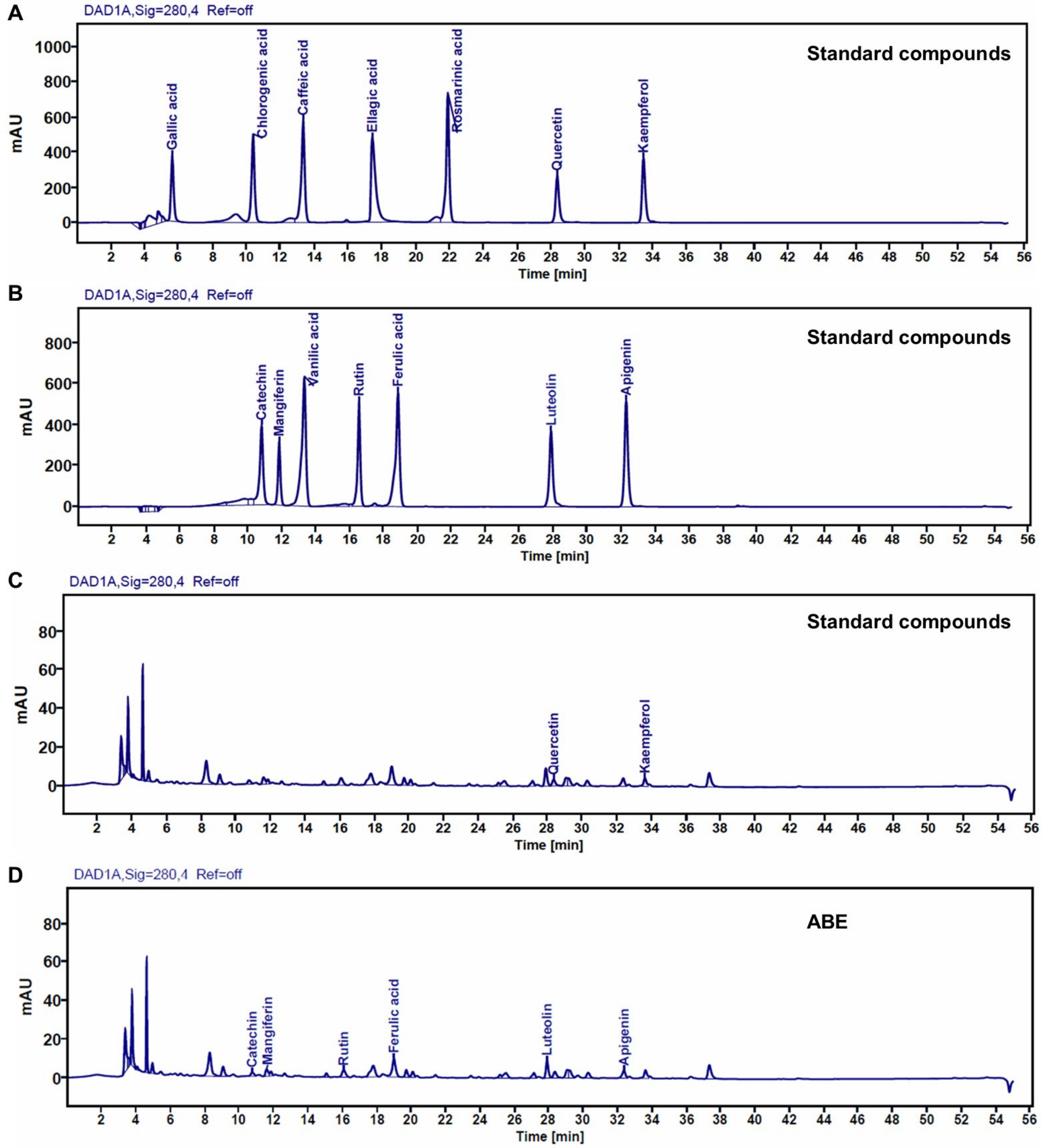

Supplementary Figure S1. The HPLC chromatograms of standard gallic acid, chlorogenic acid, caffeic acid, ellagic acid, rosmarinic acid, quercetin, and kaempferol (A), catechin, mangiferin, vanillic acid, rutin, ferulic acid, luteolin, and apigenin (B). Quercetin and kaempferol (C). ABE extract shown the retention time similarly with catechin, mangiferin, rutin, ferulic acid, luteolin, and apigenin (D).

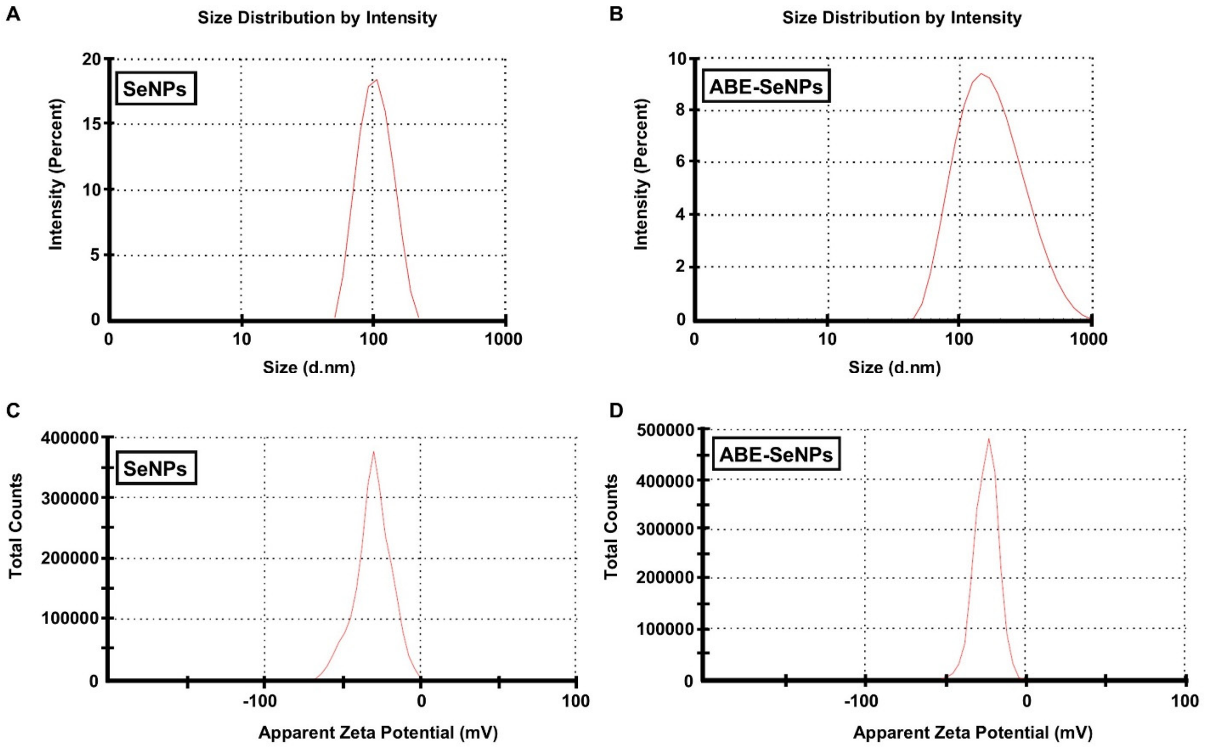

Supplementary Figure S2. Size and zeta potential of the nanoparticle were measured by dynamic light scattering. Size distribution diagram of SeNPs (A) and ABE-SeNPs (B). Zeta potential diagram of SeNPs (C) and ABE-SeNPs (D)

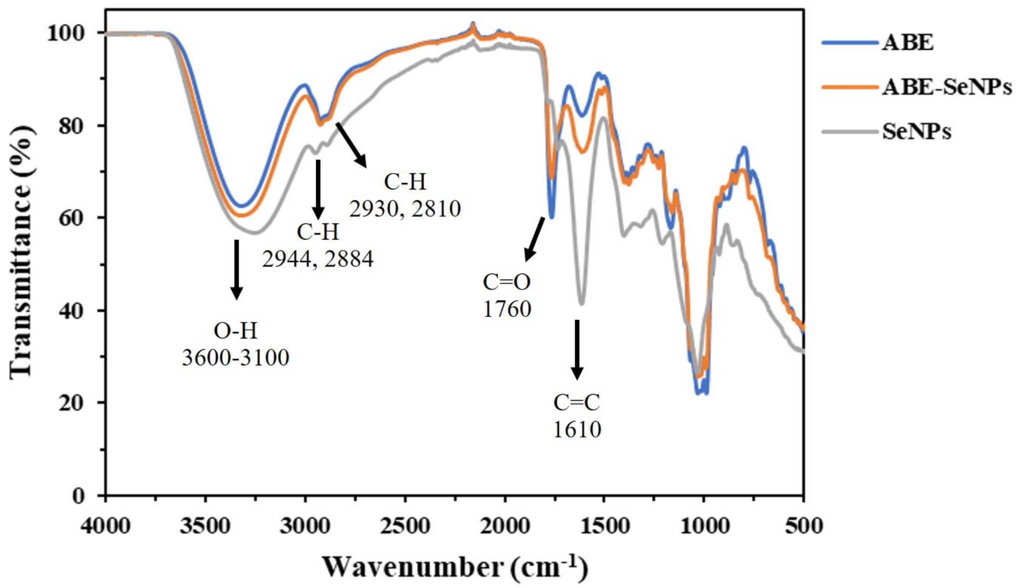

Supplementary Figure S3. FTIR Spectra of ABE, SeNPs and ABE-SeNPs. Each sample was dried and then ground into a homogeneous powder to record the infrared spectra on a Thermo Scientific Nicolet iS50 FT-IR spectrometer with a built-in diamond attenuated total reflection (ATR). Peaks at 3600-3100 (OH group), 2944, 2884 and 2930, 2810 (aliphatic C-H), 1760 (carbonyl C=O stretch), 1610 (C=C stretch) were observed.

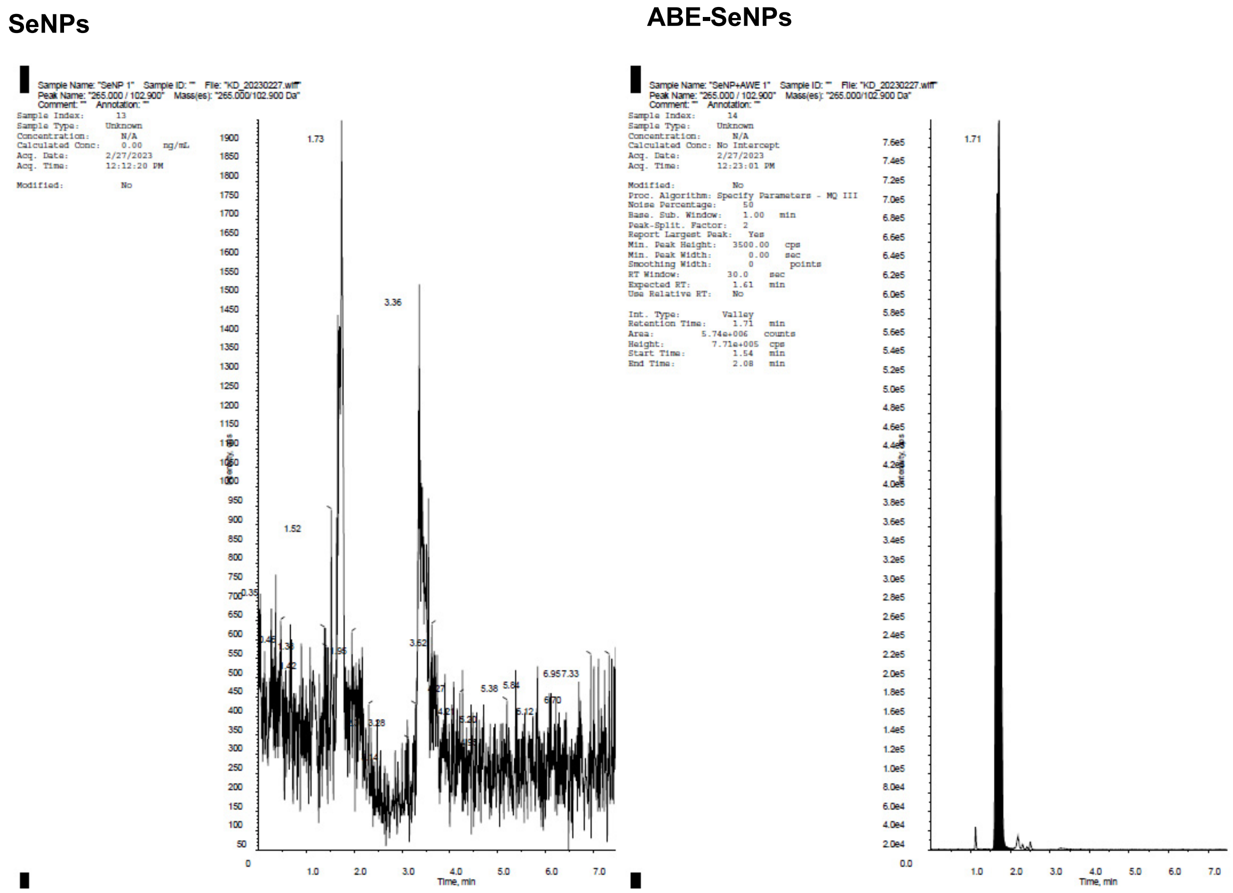

Supplementary Figure S4. LC/MS-MS Chromatography of SeNPs and ABE-SeNPs. ABE-SeNPs were extracted with methanol (ratio 1:1) to obtain ABE, the mixture then was centrifuged at 12000 rpm for three minutes. Thereafter, 200 µL of supernatant was transferred to a clean vial and used for LC-MS/MS analysis. A single peak of kinsenoside was detected only in ABE-SeNPs.

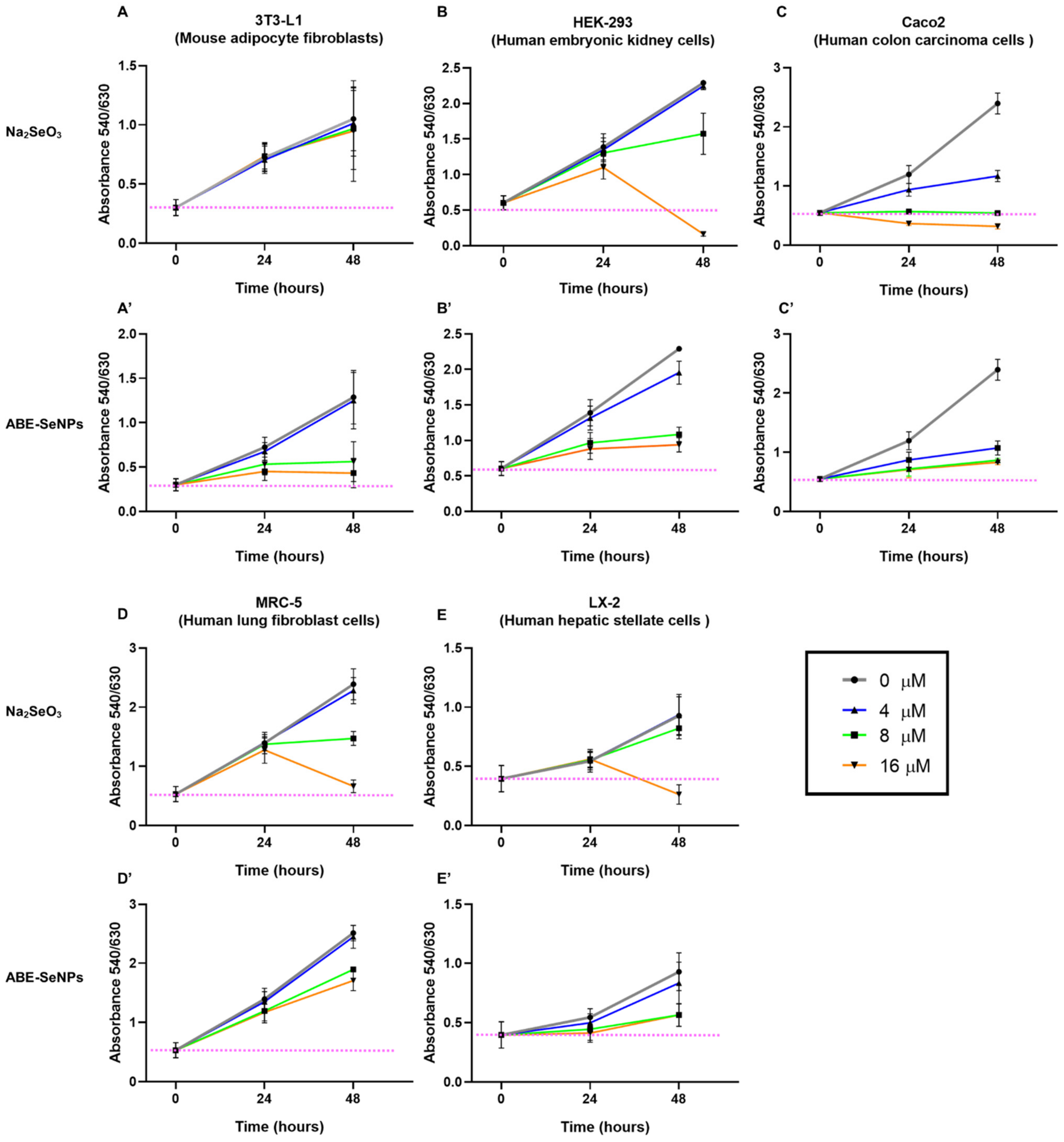

Supplementary Figure S5. Effect of Sodium Selenite (A-E) and ABE-SeNPs (A'-E') on Cell Proliferation of 3T3-L1, HEK-293, Caco2, MRC-5, and LX-2. Cell proliferation was determined using an MTT assay at indicated time. The treated cells were incubated with 0.5 mg/mL MTT solution with

serum free-medium for two hours (formazan crystal formation). The solution was gently removed, then the crystal was dissolved by dimethyl sulfoxide (DMSO). The cell viability is directly related to the absorbance of formazan. The absorbance of the colored solution was measured at 540 nm by a microplate reader. The cell number of ABE-SeNPs treated cells was increased compared to the starting point of the treatment. In addition, the growth of the treated cells was slowly increased when compared to the control, suggesting that ABE-SeNPs inhibited the proliferation of 3T3-L1, HEK-293, and Caco2 (A'-C') leading to a decrease in cell numbers. Meanwhile, sodium selenite showed a killing effect on HEK-293, Caco2, MRC-5, and LX-2 represented by the reduction of the cell number after the treatment (48 h) compared to the starting point (0 h) (B-E).

Supplementary Table S1. Total Phenolic, Kinsenoside, and Phenolic profile of ABE

Sample	Total phenolic content		Kinsenoside (μg/mg extract)	Phenolic Compounds (mg/g extract)		
	(mg GAE/g extract)	(mg FAE/g extract)		Catechin	Ferulic acid	Rutin
ABE	11.18 ± 0.74	23.28 ± 1.09	371 ± 36.75	2.54 ± 0.20	1.74 ± 0.10	2.36 ± 0.10

The data indicated as mean ±SD, n=3
